# A Case of Recurrent Acute Anterior Uveitis After the Administration of COVID-19 Vaccine

**DOI:** 10.7759/cureus.22911

**Published:** 2022-03-07

**Authors:** Manal A Alhamazani, Wejdan S Alruwaili, Bandar Alshammri, Sarah Alrashidi, Jluwi Almasaud

**Affiliations:** 1 Ophthalmology, King Khalid Hospital, Hail, SAU

**Keywords:** coronavirus, ocular manifestation, covid-19 infection, vaccination, uveitis

## Abstract

We present a case of a 37-year-old healthy man who developed acute anterior uveitis after receiving the first and second doses of the Pfizer-BioNTech coronavirus disease 2019 (COVID-19) vaccine. To the best of our knowledge, this is the first report of a recurring incidence of ocular side effects associated with COVID-19 immunization. Based on the timing of the start of symptoms with the first and second vaccinations, the absence of prior medical conditions, and unremarkable investigations, we believe that the patient's anterior uveitis may have been induced by the vaccine itself. This case suggests that vaccination could be a risk factor in uveitis development and recurrence following redosing. As a result, we recommend that ophthalmologists investigate the recent immunization status in each case of uveitis with a temporal association with COVID-19 vaccine administration and record these cases to improve the quality of data tracking of potential adverse responses to vaccines.

## Introduction

Vaccination is one of the most significant achievements in public health. It has helped reduce the incidence rate of several infectious diseases [1,2]. However, the benefits of vaccinations are accompanied by the possibility of a wide range of adverse effects [1]. In December 2020, the United States Food and Drug Administration (FDA) authorized the Pfizer-BioNTech coronavirus disease 2019 (COVID-19) vaccine for public use after demonstrating its high degree of safety, efficacy, and manufacturing quality to protect against COVID-19 [3]. The safety profile of this vaccine appears to be similar to that of other viral vaccines, with mild to severe pain, swelling, redness at the injection site, chills, fatigue, and headache being the most common side effects [4]. Although various ocular manifestations have been documented following administration of several other vaccines, there have been few reports on the ocular side effects of COVID-19 vaccination, including zoster ophthalmicus, facial nerve palsy/Bell’s palsy, abducens nerve palsy, episcleritis, scleritis, uveitis, corneal graft rejection, Vogt-Koyanagi-Harada (VKH) disease reactivation, acute macular neuroretinopathy, acute middle maculopathy, subretinal fluid, superior ophthalmic vein thrombosis, central serous chorioretinopathy, and the onset of Graves’ disease [5-7]. Here, we report a unique case of anterior uveitis following COVID-19 vaccine administration.

## Case presentation

This study followed the tenets of the Declaration of Helsinki and written consent was obtained from the patient. Since this case report did not include any patient identifying information, it does not require the Institutional Review Board approval.

A 37-year-old man presented to the ophthalmology department of our institution on August 8, 2021, with a complaint of photophobia, redness, a decrease of vision, and pain in the left eye that began three days after receiving the second dose of the Pfizer-BioNTech COVID-19 vaccine.

**Figure 1:**
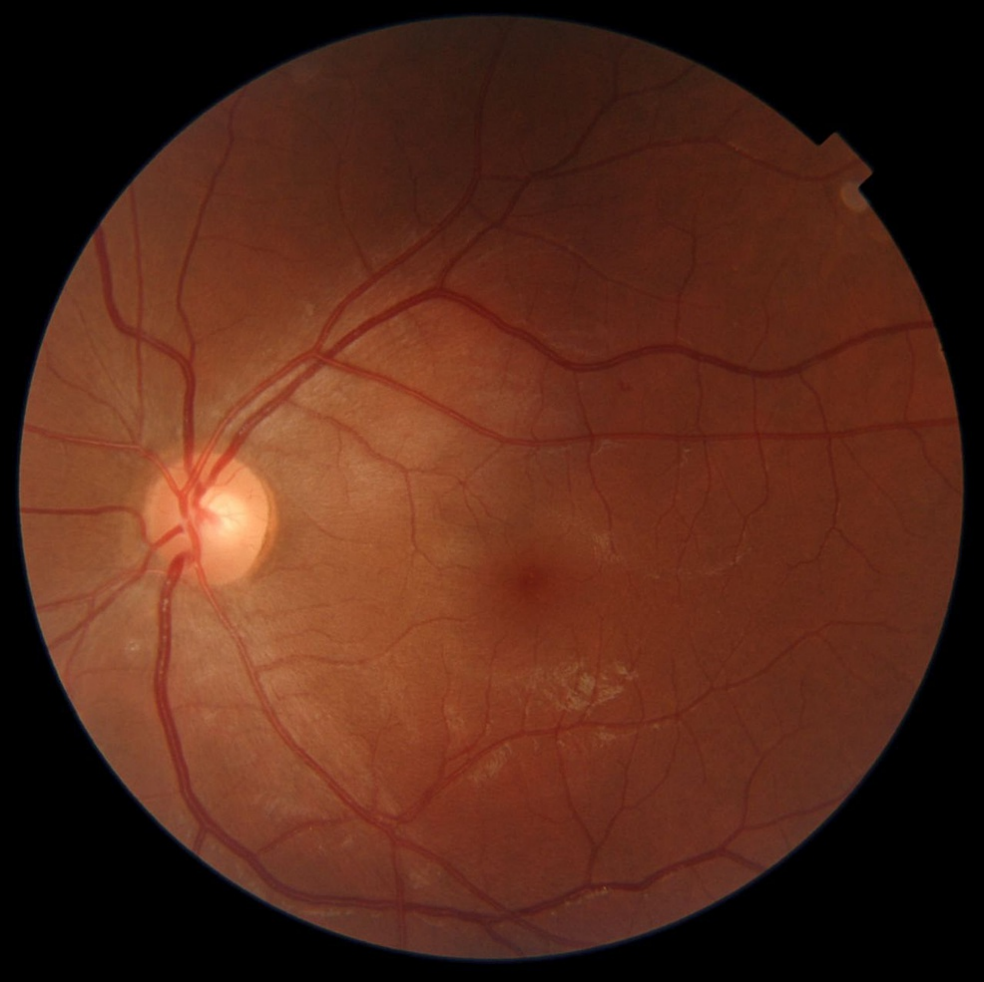
Color fundus photography shows no abnormal finding.

**Figure 2:**
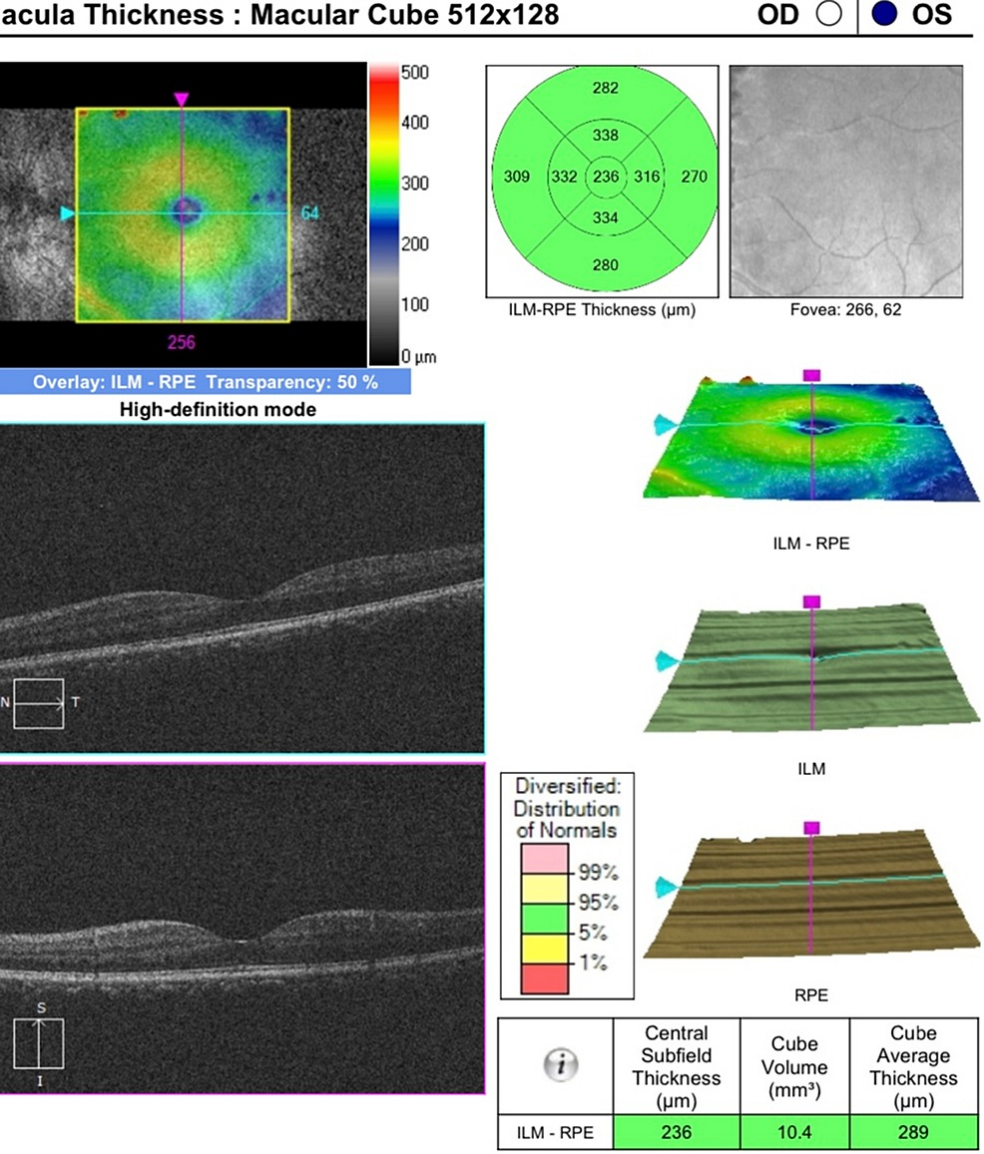
Optical coherence tomography shows no macular edema in the left eye. ILM, internal limiting membrane; RPE, retinal pigment epithelium; OD, oculus dexter (right eye); OS, oculus sinister (left eye).

Upon examination, his best-corrected visual acuity was found to be 20/20 in the right eye and 20/70 in the left eye. The intraocular pressure in both eyes was within the normal range. Slit-lamp examination of the left eye showed intense ciliary flush and a clear cornea with no keratic precipitates. The anterior chamber contained 4+ cells and 2+ ﬂare. Posterior synechiae were also observed. The pupils were reactive to light, the lens was clear, and the anterior vitreous was normal. Fundoscopic examination revealed normal findings, and there was no evidence of vasculitis, choroiditis, or retinitis (Figure 1). Cystoid macular edema was ruled out using optical coherence tomography (Figure 2). The right eye examination was normal.

The patient’s medical, ocular, familial, and medication history was unremarkable, and he denied any joint pain, bowel disease, or oral and genital ulcers. However, he had one episode of similar presentation and intensity in the same eye after receiving the first dose of the Pfizer-BioNTech COVID-19 vaccine on June 22, 2021. He had then been treated in our institution for acute anterior uveitis and had fully recovered with the treatment.

**Figure 3:**
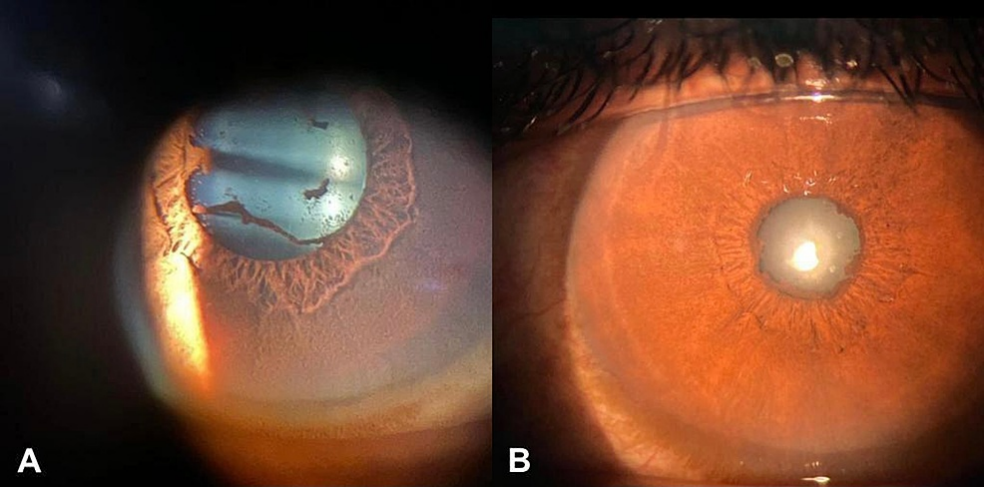
Ocular findings of anterior acute uveitis after administration of COVID-19 vaccine (A) and slit-lamp photographs show mild vascular engorgement of the bulbar conjunctiva and posterior synechiae (B).

At the visit, the patient was diagnosed with recurrent acute anterior uveitis. Since the uveitis recurred, he was also referred for imaging and laboratory workup to explore possible systemic causes. He had normal complete blood counts, and his erythrocyte differentiation and sedimentation rates were also normal. Blood biochemical results were within normal limits. The tuberculin test, as well as venereal disease research laboratory, rheumatoid factor, and antinuclear antibody tests, yielded negative results. His angiotensin-converting enzyme level, which was examined to rule out sarcoidosis, was normal, and examinations of stool and urine did not reveal abnormal findings. The patient was negative for toxoplasma IgG antibodies, and chest and spinal radiography revealed normal findings. Topical prednisolone acetate 1% and cyclopentolate were administered to the patient. In two days of follow-up, his symptoms and clinical signs improved compared to the initial visit with mild bulbar conjunctiva congestion and posterior synechiae (Figure 3). In the first week of follow-up, his visual acuity had improved, no cells or flares were observed in the anterior chamber, and there was no further sign of inflammation.

## Discussion

Uveitis is an ophthalmic emergency characterized by the inflammation of the middle tissue, or uvea, of the globe of the eye. Despite its rarity, vaccine-induced uveitis has been reported in several cases, including due to bacillus Calmette-Guérin (BCG), diphtheria, tetanus, and pertussis (DPT), measles, mumps, and rubella (MMR), influenza, varicella, and smallpox vaccines [1]. Here, we present a case of two episodes of acute anterior uveitis that developed shortly after immunization with the Pfizer-BioNTech mRNA COVID-19 vaccine. To the best of our knowledge, this is the first report of recurrent incidence of ocular side effects associated with COVID-19 vaccination.

In this patient, the first episode started in the left eye, five days following the vaccination with symptoms of photophobia, redness, pain, and decrease of vision. The patient was diagnosed with acute anterior uveitis with a possible association to COVID-19 vaccination. Full recovery was seen after the first week of follow-up with topical prednisolone acetate 1% four times daily and cyclopentolate three times daily. Hence, a tapering prednisolone acetate regime was started with the discontinuation of cyclopentolate. Six weeks later, the patient returned to the ophthalmology department in our institution with similar symptoms of the first episode of anterior uveitis after receiving the second dose of the COVID-19 vaccine. Interestingly, the symptoms started earlier than during the first episode; however, the response to the treatment was similar. Since the uveitis recurred, all known contributing factors and disease associations with uveitis were excluded through a case history. Additionally, the necessary imaging and laboratory workup to explore the possible systemic causes showed normal findings. Besides the present case, a recent review of COVID-19 vaccine-associated uveitis identified in five cases and one multicenter retrospective case series reported that in most of the patients, the ocular manifestation of uveitis appeared after receiving the second dose of different types of COVID-19 vaccines. In most of these cases, patients received the Pfizer-BioNTech mRNA vaccine, with a mean time of 7.5 ± 7.3 days (1-30 days) from vaccination to uveitis onset, which showed full resolution in most of the cases with adequate treatment. Only two cases showed significant improvement [6]. Although the pathophysiology of postimmunization vaccine-induced uveitis is unclear, it is thought to be linked to mRNA vaccine-induced cellular and humoral immune responses, which can lead to molecular mimicry and immunological cross-reactivity, potentially leading to autoimmune disease [8].

In summary, consideration of vaccination as a potential cause of uveitis cannot be completely ruled out, especially with the rising number of recent uveitis cases following vaccination. The temporal relationship between immunization and symptom onset of uveitis in both episodes in our case suggests that this may not be a coincidence. Instead, the recurrence of the unusual adverse effects following redosing in our case can be seen as a positive rechallenge that provides evidence while evaluating the causality in cases of uveitis. In our case, post-COVID-19 vaccine ocular inflammation in the first episode and its recurrence in the second episode was temporary and resolved with topical therapy without any complications or prolongation. Therefore, we recommend receiving the COVID-19 vaccination and suggest postponing vaccination if the patients have active eye inflammation. Notwithstanding the case report presented here, further studies are warranted to understand the pathophysiology and high-risk characteristics of patients who are susceptible to developing uveitis post-immunization.

## Conclusions

The present case study suggests that vaccination may be a risk factor for uveitis development and recurrence following redosing. As a result, we recommend that ophthalmologists investigate the recent immunization status in each case of uveitis with a temporal association with COVID-19 vaccine administration and record these cases using the Vaccine Adverse Event Reporting System by the FDA to improve the quality of data tracking of potential adverse responses associated with vaccines.
